# Similar usage of T‐cell receptor β‐chain between tumor and adjacent normal tissue in hepatocellular carcinoma

**DOI:** 10.1002/cam4.70121

**Published:** 2024-08-27

**Authors:** Jie‐Zuan Yang, Shao‐Yan Xu, Dang‐Sheng Xiao, Jin‐You Li, Xiu‐Yuan Jin, Dong Yan

**Affiliations:** ^1^ The First Affiliated Hospital, Zhejiang University School of Medicine, State Key Laboratory for Diagnosis and Treatment of Infectious Diseases, National Clinical Research Center for Infectious Diseases Hangzhou China; ^2^ Division of Hepatobiliary and Pancreatic Surgery, Department of Surgery The Second Affiliated Hospital of Zhejiang University School of Medicine Hangzhou China; ^3^ Key Laboratory of Diagnosis and Treatment of Aging and Physic‐Chemical Injury Diseases of Zhejiang Province, The First Affiliated Hospital Zhejiang University School of Medicine Hangzhou China

**Keywords:** complementarity determining region 3, hepatocellular carcinoma, immune privilege, immune repertoire, T‐cell receptor β‐chain

## Abstract

**Background:**

In this study, we comprehensively profiled the T‐cell receptor (TCR) repertoire of the tumor and adjacent normal tissue in patients with HBV‐associated hepatocellular carcinoma (HCC) and determined the baseline characteristics and clinical significance of TCR.

**Methods:**

High‐throughput sequencing was used to determine the profile of complementarity‐determining region 3 (CDR3) of the TCR‐β chain variable (TRBV) in the tumor and normal tissue samples of 14 HCC patients. At the same time, TRBV diversity and differences in expression between tumor and normal tissues were investigated. The cumulative frequency of top 100 CDR3 (CF100), clonality, and Shannon entropy as indices to evaluate diversity,

**Results:**

The diversity of TRBV CDR3 showed no significant difference between tumor and normal tissues. Of the 58 V gene segments in TRBV, TRBV16 and TRBV7‐6 had a significantly higher frequency in the tumor group than in the normal group (*p* < 0.05). The frequency of 14 J gene segments showed no significant difference between tumor and normal tissues. In contrast, the frequency of 22 TRBVx/BJx combinations was significantly higher in the tumor than in the normal tissue. In addition, the length and type of TRBV CDR3 were similar in tumor and normal tissues, and a Gaussian distribution was observed in both groups.

**Conclusion:**

This study provided a large amount of information about the TCR lineage in HBV‐associated HCC, laying the foundation for further research. In addition, the fact that the immune repertoire (TRBV CDR3) hardly differs between tumor and adjacent normal tissue provides a new clue for exploring the mechanism of the liver as an organ with immune privileges.

## 1 INTRODUCTION

Primary hepatic carcinoma is one of the most common malignancies in the world,^1^ which mainly includes hepatocellular carcinoma (HCC), intrahepatic cholangiocarcinoma (ICC), fibroblast carcinoma, and hepatoblastoma, and has become the second most deadly cancer worldwide, with HCC being the most common primary liver carcinoma.^2^ Hepatocellular carcinoma is characterized by an insidious onset, difficult early diagnosis, rapid progression, and a high rate of recurrence and metastasis. Routine treatment modalities include surgical resection, chemotherapy, radiotherapy, radiofrequency ablation, vascular embolization, or liver transplantation.^3^ Apart from the fact that early small hepatic carcinoma may benefit from surgical resection, there is still no effective treatment approach for advanced HCC.^4^ However, liver transplantation is often considered the most effective treatment for end‐stage liver disease. In clinical practice, the intensity of rejection between host and graft after liver transplantation is usually low, and the liver is considered an immune‐privileged organ whose mechanism is poorly characterized.^5^ In addition, it is still urgent to explore the pathogenesis of liver cancer and to develop a diagnosis and treatment method for this type of malignant disease.^6^

The development and prognosis of HBV‐associated HCC are closely related to the cellular immune response mediated by T lymphocytes.^7^ Infiltrating T lymphocytes are not only antitumor effector cells but also the most important effector cells in the host lesion and play an important role in tumorigenesis and development.^8^ The study of the TCR immune repertoire on the cell membrane can help us to better understand the functional principles of the immune system and its applications in disease and immunotherapy.^9^ Complementary determining region‐3 (CDR3) of the TCR exhibits the greatest variation and is a highly variable region that best represents the response characteristics of T cells.^10^ Previous studies have shown that the TCR repertoire can reflect the diversity of T cells in the body during a given period and the ability of the host immune system to respond to external stimuli.^7^, ^8^ Analysis of TCR libraries can assess the diversity of T‐cell clones in tumor tissues,^11^ reflecting the ability of the immune system to fight tumors and providing the basis for TCR T cells to treat tumors, including the development of vaccines. By comparing the composition of T cell clones in tumor tissue and normal tissue, the activity level of the immune response to the tumor can be understood.^12^

T‐cell receptor sequencing (TCR seq) uses multiplex polymerase chain reaction (PCR) or rapid amplification of the 5'‐cDNA end (5'‐RACE) to amplify CDR3. In recent years, this method has often been used in combination with high‐throughput sequencing technology (HTS) to assess the diversity of the TCR repertoire.^13^ At the same time, many researchers have focused on the diversity of the TCR repertoire, tumor status, and tumor immunotherapy and have shown their close relationships, such as in malignant melanoma and in patients with advanced lung cancer.^14^, ^15^ However, there are only a few research projects and applications of the TCR repertoire analysis technique in the liver itself as an immune‐privileged organ and HBV‐associated HCC.^16^

## 2 MATERIALS AND METHODS

### 2.1 Patients and tissue samples

Tissue samples were collected between May and December 2022. Samples of tumor and adjacent normal tissue (normal tissue) were collected from 14 patients diagnosed with primary HCC histopathologically (immunohistochemical testing as additional evidence, if needed) and treated with surgical resection at the Second Affiliate Hospital, Zhejiang University School of Medicine (Hangzhou, China). The 14 patients had not been treated before surgery and had no other immune‐related diseases, infectious diseases, autoimmune diseases, or other tumors. In addition, all tissue samples were taken from 14 patients with HCC. In 8 patients both tumor and adjacent tissue was sampled, in 5 patients only tumor tissue was sampled and in one patient only adjacent tissue was sampled. Therefore, a total of 13 (8 + 5) tumor tissues and 9 (8 + 1) adjacent (normal tissues were used for the study) were independently confirmed by at least two experienced pathologists. Considering the spatial heterogeneity of tumor tissue, three different sites were taken from each tissue sample and mixed together to add TRIzol (Invitrogen, USA). The tissue lysates were immediately stored at −70°C until further processing. This study was approved by the Ethics Committee of the Second Affiliate Hospital, Zhejiang University School of Medicine (SHZJU), and is in accordance with the Declaration of Helsinki (2018). Participants agreed and signed an informed consent form.

### 2.2 TRBV gene amplification and high throughput sequencing of CDR3

Total RNA was extracted from 1 mL of tissue lysate by the general method according to the guidelines for the use of reagents. Total RNA was reverse transcribed into cDNA using the iScript cDNA Synthesis Kit (Bio‐Rad Inc., Hercules, USA) according to the manufacturer's instructions. For the generation of TRBV sequencing libraries, nested amplicon arm PCR was performed in two rounds using a Multiplex PCR Assay Kit ver. 2 (TaKaRa, Dalian, China) with specific primers against each variable and constant gene, the detailed steps were described in our previous report.^17^ In addition, PCR products were purified by agarose gel electrophoresis, amplified using Illumina sequencing primers with different sample barcodes, and subjected to high‐throughput sequencing using the Illumina HiSeq X Ten platform (Illumina, San Diego, CA), sequenced, and analyzed.

### 2.3 TRBV sequence analysis

The raw sequencing data were uploaded and subjected to adaptor trimming, and reads containing more than 8 ambiguous bases (N) or poor quality (15% nucleotide positions with a Phred quality <30) were removed by a custom script in Perl. The high‐quality reads were used for subsequent analysis. The V, D, and J genes were identified using BLAST+ (version 2.7.1) by aligning them to their reference sequences in the ImMunoGeneTics (IMGT: http://www.imgt.org/) international information system. The CDR3 sequence was defined as the amino acids between the second cysteine of the V region and the conserved phenylalanine of the J region. T‐cell receptor algorithms include the TCR recognition algorithm, signal transduction pathway regulation algorithm, etc. The key technologies of TCR include the application of machine learning, deep learning, and other algorithms, as well as the application of computer simulation and simulation technologies.^18^, ^19^

Finally, the number of V, V‐J, and VDJ genes and the diversity index, such as Shannon entropy (SE), clonality, Simpson, CF100, and D50, were calculated based on existing formulas. In addition, the frequency of TRBV, TRBJ, and TRBV‐BJ was also calculated. The heatmap of VJ gene linkage was created using the online tool (http://www.ehbio.com/ImageGP/), the CDR3 length distribution and the distribution of CDR3 and VDJ numbers were calculated.

### 2.4 Statistical analysis

Statistical analyses were performed using the Prism package, version 8.0 (GraphPad Software Inc, San Diego, USA). Data with normal distribution were expressed as mean ± SD. The difference between the two groups was determined using a t test (with two tails) when the data were normally distributed and the variance was equal. The Mann–Whitney test was used for comparison between different groups with nonnormal distribution. The correlation between TCR diversity and clinical indicators was determined using the Spearman rank test. *p*‐values of <0.05 were considered significant.

## 3 RESULTS

The clinical and biochemical characteristics of 14 patients with HBV‐associated HCC enrolled in this study are shown in Table 1. Three patients were female (21.4%), and the median age was 56 years (range 39–80 years). According to the staging system of the Union for International Cancer Control (UICC, 2002), a total of 5 (35.7%) patients were in stage I, 4 (28.6%) patients in stage II, and 5 (35.7%) patients in stage III. In addition, all 14 patients had no lymph node metastases (N0) or distant metastases (M0). Moreover, 2 (14.3%) of these patients had recurrences or metastases in the following 6 months of follow‐up.

**TABLE 1:** Clinical and biochemical indices of the 14 patients with HBV‐associated HCC.

Characteristics	No. (%)
Age (years)	
Median (range)	56 (39–80)
Gender	
Male	11 (78.6%)
Female	3 (21.4%)
Tumor (T) stage	
T1	5 (35.7%)
T2	4 (28.6%)
T3 (A + B)	5 (35.7%)
Lymphoid nodal (N) status	
N0	14 (100%)
Distant metastasis (M) status	
M0	14 (100%)
TNM stage	
I	5 (35.7%)
II	4 (28.6%)
III (A + B)	5 (35.7%)
HBV	
Positive	14 (100%)
AFP (ng/mL)	
Median (range)	25.95 (0.7–586.1)

### 3.1 Gene amplification and CDR3 diversity

Sequence profiles of the TCR‐β‐chain variable region (TRBV) in tumors and adjacent normal tissues (normal) from 14 HBV‐associated HCC patients were obtained and are shown in Figure S1. The average number of raw reads and QC (quality control) reads are 5,201,836,692 and 4,091,600,771 in tumor and normal tissues are 5,327,619,444 and 4,300,981,111, respectively. The average number of productive unique CDR3aa sequences was 8912.46 and 7191.56 per sample in tumor and normal tissue, respectively.

**FIGURE 1:**
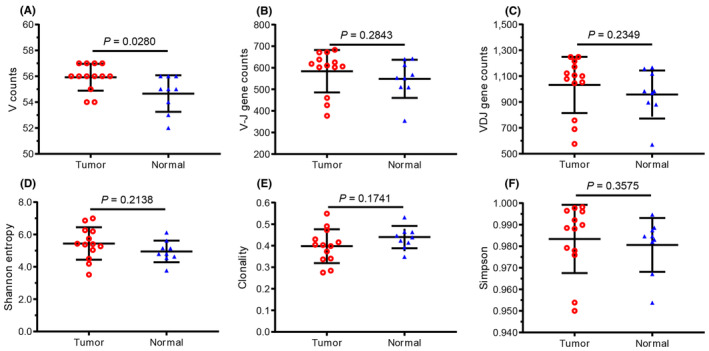
Comparison of the number of V, V–J, and VDJ genes, and their diversity indices between tumor and adjacent normal tissue. (A) V gene, (B) V–J gene combination, (C) VDJ gene combination, (D) Shannon Entropy (SE), (E) Clonality, and (F) Simpson, of tumor and adjacent normal tissue (normal) for each patient. Each dot represents an index of each patient, and the bars show the mean ± standard deviation (SD). The tumor and adjacent normal tissue (normal) were harvested from 14 patients with HCC, the paired liver tumor tissue (tumor), and normal tissue from 8 patients, only the tumor from 5 patients, and only the normal tissue from 1 patient. A total of 13 (8 + 5) tumor tissues and 9 (8 + 1) normal tissues were used for the study.

For the specific V gene segments, the average number of V genes was 54.62 and 52.9 in tumor and normal samples, respectively, with a significant difference between them (Figure 1A). In addition, 48 V gene segments and 14 J gene segments were identified in most samples, resulting in an average of 583.85 different V–J pairs from 13 tumor samples and 548.8 different V–J pairs from 9 normal samples (Figure 1B). Comparison of the number of VDJ gene combinations revealed no significant difference between tumor and normal samples (Figure 1C). There was also no significant difference between tumor and normal samples in diversity indices (SE, Clonality, Simpson) based on CDR3aa frequencies (Figure 1D–F).

### 3.2 Similar CDR3 profile between tumor and adjacent normal tissue

**FIGURE 2:**
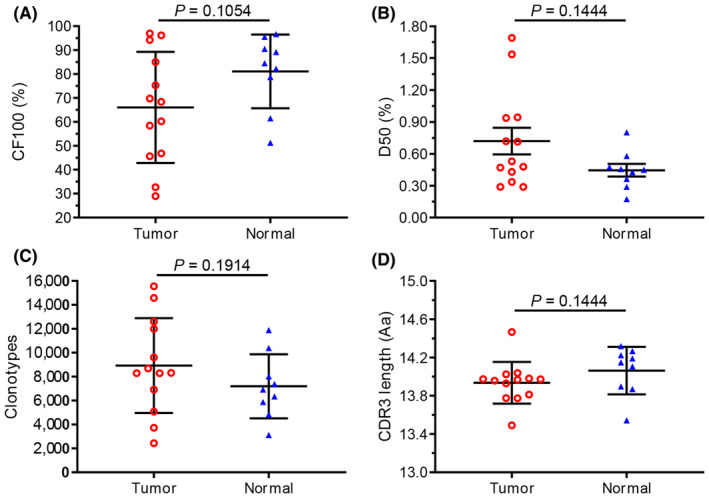
Comparison of cumulative frequency, number of clonotypes, and CDR3 length between tumor and adjacent normal tissue in HCC patients. (A) Cumulative frequency of top 100 CDR3s (CF100) in tumor and adjacent normal tissue (normal). Data points represent CF100s in the total repertoire of each patient, and bars show the mean (±SD) of CF100s. (B) Clonal amplification of each patient's TRBV clonotype was described by D50. (C) Comparison of the number of CDR3 clonotypes between tumor and normals. (D) Comparison of the average CDR3 length between tumor and normals. Data points represent the average CDR3 length of each individual, and bars show the mean (±SD) of CDR3 length. D50, the ratio between the number of unique CDR3 accounting for 50% of the total reads and the total number of unique CDR3 reads. CDR3, complementary determinant region 3; HCC, hepatocellular carcinoma; TRBV, TCR beta chain variable.

The CDR3 profile was also represented by its diversity indices. For example, there was no significant difference in CF100 between the tumor and adjacent normal tissue (normal) in HCC subjects (Figure 2A), similar results were also found in two other diversity indicators (D50, Clonotypes) (Figure 2B,C). Moreover, the average CDR3aa length in each sample was about 14 AA (amino acid), and there was no significant difference between tumor and normal tissues (Figure 2D). At the same time, the distribution of CDR3aa length in both tumor and normal tissues is a normal distribution (Gaussian distribution) (Figure S2).

### 3.3 CDR3/VDJ number in different distribution regions

The number of CDR3 in different distribution regions was compared between tumor and normal tissues, and a significant difference was found only in the range of 0.01% to 0.1% (Figure S3A). Also, no significant difference in the number of VDJ was found between tumor and normal tissues for each distribution region (Figure S3B).

### 3.4 Frequencies and comparison of V, J, and V–J pairs

**FIGURE 3:**
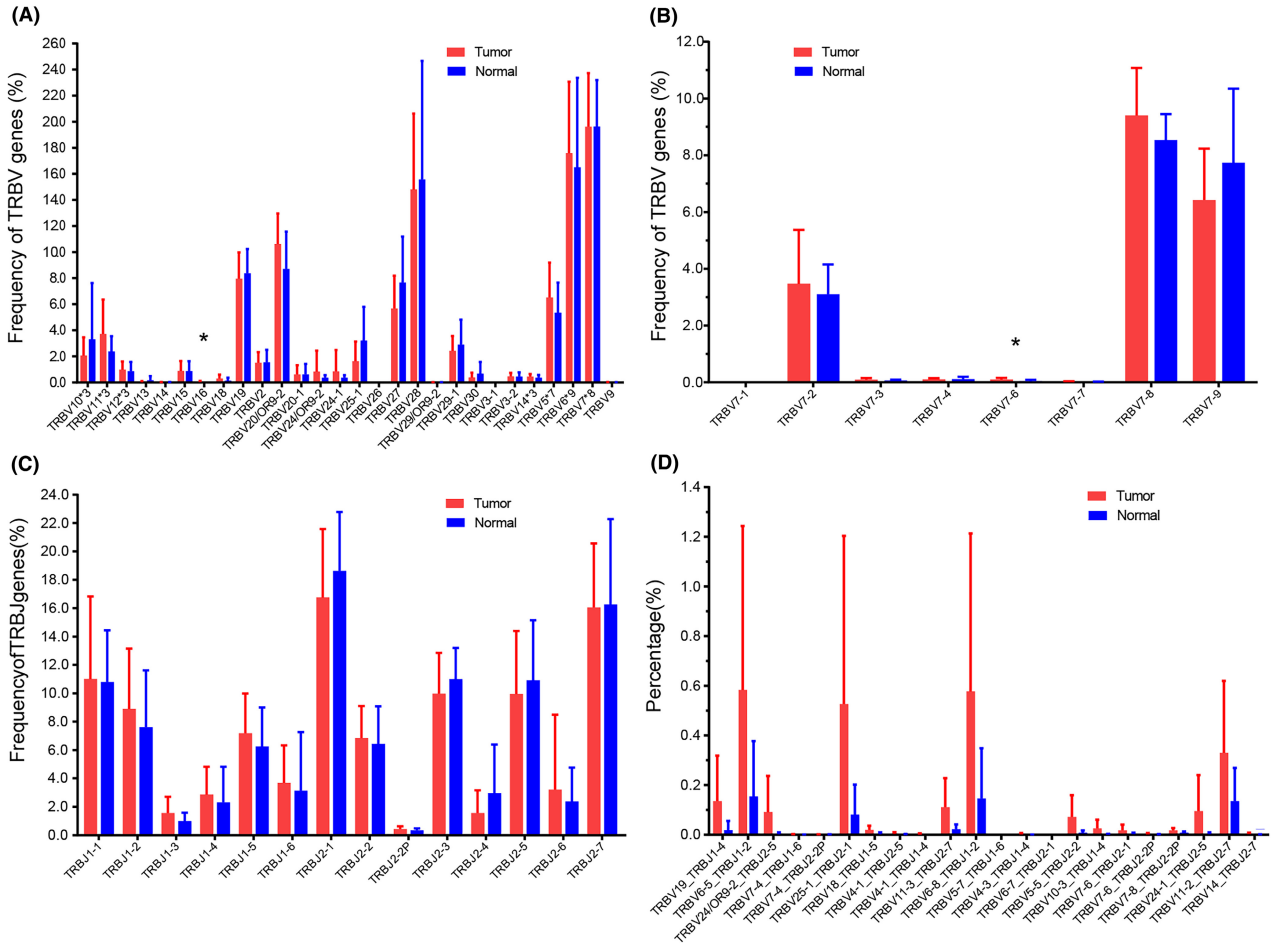
Comparison of the utilization of the paired genes TRBV, BJ, and BV/BJ in tumor and adjacent normal tissue. (A) The usage frequencies of 28 TRBV gene subfamilies in tumor and adjacent normal tissues (normal). The number after “*” represents the number of subfamilies in that TRBV family, for example, TRBV7*8 means that the TRBV7 family includes 8 subfamilies. (B) The 8 subfamilies of TRBV7 in detail. (C) The usage frequencies of 14 BJ genes in two tissues. (D) The usage frequencies of 22 TRBV/BJ genes that are particularly abundant in tumor tissues compared with normal tissues. Data show the mean (±SD) abundance for each subject. Data were compared using the Mann–Whitney test, *p < 0.05. Of all TRBV families, only TRBV16 and BV7‐6 show significantly different expression between the two tissues (*p* = 0.0443, 0.0228). For TRBJ families, there is no significant difference between the two tissues. TRBV, TCR beta chain variable.

As for TRBV gene families, 58 BV subfamilies were classified into 28 BV families, and the details of 8 genes of BV7 subfamily were shown (Figure 3A,B). Among the 58 BV subfamilies, TRBV16 and BV7‐6 showed a significantly higher percentage of tumors than in normals. In addition, there was no significant difference in expression between tumor and normals in any of the 14 TRBJ subfamilies (Figure 3C). However, there are 22 TRBVx/BJx with significantly higher expression in tumors compared to normal tumors (Figure 3D), and more details are shown in Table S1.

### 3.5 Characteristics of V–J gene combinations

**FIGURE 4:**
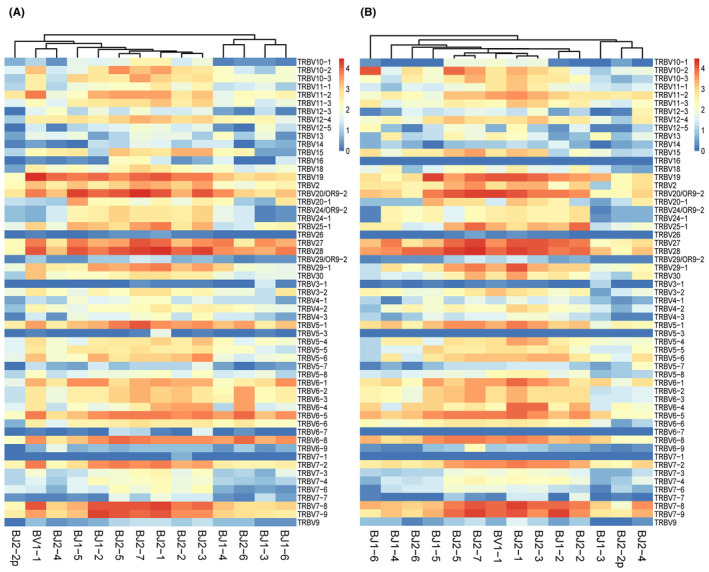
Expression frequency of TRBV/BJ combination in tumor and adjacent normal tissues. (A) Heatmap of BV/BJ gene combinations in HCC and (B) adjacent normal tissues (normal) from IMGT/Stat clonotype analysis shows that there are some different BV/BJ gene combinations between tumor and normal tissues. Thus, the 14 TRBJ families in tumor and normal tissues are obviously divided into two clusters, with each cluster consisting of different TRBJ families. The heatmap bar shows the frequency of use of BV/BJ gene combinations in each sample. TRBV, TCR beta chain variable.

We evaluated the gene usage of 58 BVs and 14 BJs combinations (58 × 14 = 812) for tumor and adjacent normal tissue, and overall, no clear classification of the TRBV family between tumor and normal tissue was apparent. However, the composition of TRBJ family clusters differed between the two groups, for example, there were 2 BJ clusters in tumor tissue (Figure 4A), C1: BJ1‐6, BJ1‐3, BJ2‐6, BJ1‐4; C2: BJ2‐3, BJ2‐2, BJ2‐1, BJ2‐7, BJ2‐5, BJ1‐2, BJ1‐5, BJ2‐4, BJ1‐1, BJ2‐2p, while in normal tissue (Figure 4B) the BJ clusters C1: BJ2‐4, BJ2‐2p, BJ1‐3; C2, BJ2‐2, BJ1‐2, BJ2‐3, BJ2‐1, BJ1‐1, BJ2‐7, BJ2‐5, BJ1‐5, BJ2‐6, BJ1‐4, BJ1‐6.

### 3.6 Correlation between TCR diversity and clinical indicators

**FIGURE 5:**
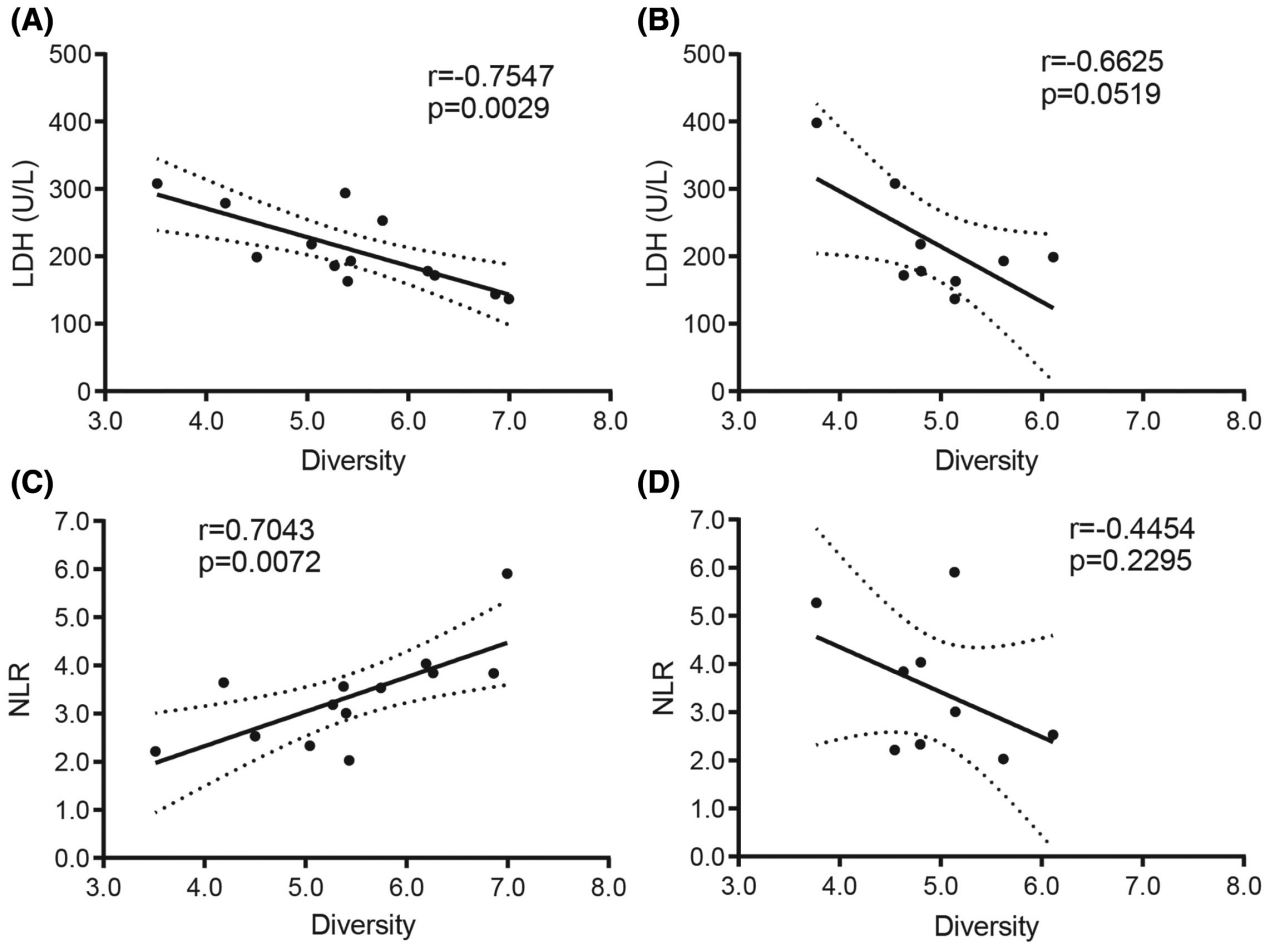
Correlation between TCR diversity and biomarkers of systemic inflammation. Correlation between TCR diversity (Shannon entropy) and LDH in (A) tumor, in (B) adjacent normal tissue (normal); correlation between TCR diversity and NLR in (C) tumor, in (D) normal. Statistical analysis was performed using the Spearman rank test. LDH, lactate dehydrogenase; NLR, neutrophil/lymphocyte ratio; TCR, T‐cell receptor.

To investigate the correlation between TCR diversity and clinical indicators, we then estimated two systemic inflammatory biomarkers, lactate dehydrogenase (LDH) and neutrophil‐to‐lymphocyte ratio (NLR), which at high levels indicate a deteriorated immune status.^20^ In the present study, we found a significant negative correlation between the diversity of CDR3 and LDH in tumor tissue (Figure 5A), but the negative correlation was not significant in normal tissue (Figure 5B). In addition, the positive correlation between the diversity of CDR3 with NLR was significant in tumor (Figure 5C), but the correlation was no significance in normal tissue (Figure 5D).

## 4 DISCUSSION

The immunological microenvironment of primary tumors is related to the occurrence and progression of the disease, as well as immunotherapy as one of the most important hotspots in the treatment of HCC, which can promote tumor‐specific immunity by activating host immune cells to fight liver tumor cells.^21^, ^22^ Generally, a large number of lymphocytes infiltrate the tumor tissue, the TCR binds to the specific antigen presented by the MHC with its CDR3 structure in the β‐chain V region. Each T cell expresses only one type of TCR CDR3, resulting in a diversity of T‐cell populations targeting different foreign antigens,^23^ and the diversity of CDR3 could represent the immune status of the host. In addition, T cells are also involved in chemotherapy and radiotherapy.^24^ Therefore, detailed analysis of T cells can provide important insights into an individual tumor and immunity.^25^

In the present study, high‐throughput sequencing technology (HTS) was used and an average of 5201836.7 and 5327619.4 QC reads were obtained from tumor and adjacent normal tissues, respectively. In addition, the length and type of tumor CDR3aa were similar to those in the normal group, and all showed a Gaussian distribution (Figure S2) in accordance with the previous report,^26^ indicating that the immune system database group was successfully established, and the results of this study were reliable.

In the present study, we used HTS to investigate the TCR lineage formed based on CDR3 diversity in HCC and adjacent normal tissue samples. The results showed that the average number of unique CDR3 in the tumor (8912.46) was higher than that in adjacent normal tissues (7191.56), indicating high TCR diversity in liver tumor tissues. In contrast to other tissues, the diversity of infiltrating lymphocytes in tissues normally decreases with invasion and stimulation by foreign antigens.^27^ Our results show that CDR3 diversity is lower in normal tissues than in tumor tissues, which may contribute to the fact that the liver is a site of immune privilege associated with a weak immune response to foreign antigens, and that this phenomenon is common in liver transplantation.^28^

To compare the difference in expression between tumor and normal tissues in HCC patients, SE, Simpson, CF100, and D50 were used as TRBV assessment indicators for β‐chain CDR3 diversity, showing that the diversity of TRBV CDR3 sequences of the tumor group had no significant difference from that of the normal group. This result differs from a previous report on another cohort of HBV‐associated HCC patients, which suggested that the CDR3 diversity of TRBV is higher in tumor tissues than in adjacent nontumor tissues.^29^ In addition, BV and BJ genes were used differently in this report. However, BV16 and BV7‐6 genes were more abundant in tumor tissues than in normal tissues in our study, and there was no difference between them in BJ. Therefore, our results are similar to the recent findings of other teams.^1^, ^16^

In general, healthy individuals with higher TCR diversity indicate adequate immune surveillance, and TCR diversity reflects the immune status of the host.^30^ Patients with low TCR diversity may have impaired immune status,^31^, ^32^ which is a potential negative predictor of immune checkpoint inhibitor treatment. In the present study, no significant difference was found between TRBV in tumor and adjacent normal tissue, although the diversity of TRBV in normal tissue was lower than in tumor. This finding may partly explain the low response to immune checkpoint therapy (such as PD‐1, PD‐L1) in liver cancer.^33^, ^34^ It is generally agreed that poor antitumor immunity may be reflected in a lower diversity of the TCR repertoire. Conversely, increased CDR3 diversity also predicted better clinical outcomes with conventional cancer therapy.^13^

Higher LDH and NLR levels indicate a worsened immune status in patients.^20^ In the present study, the correlations (CDR3 diversity vs. LDH, CDR3 diversity vs. NLR) are significant in tumor tissues, but there are no significant correlations in adjacent normal tissues. These results suggest an impaired immune status in liver tumor tissues and a relatively normal immune status in normal tissues in patients with HCC.^35^ Furthermore, in the present study, we preliminarily investigated the relationship between absolute lymphocyte count (ALC) and CDR3 diversity indicator (SE) and found a negative correlation between them in tumor tissues, while the correlation was positive in normal tissues (Figure S4). This result is consistent with previous findings and suggests that many lymphocytes are dysfunctional in liver cancer tissues.^36^, ^37^

The limitation of this study is that fewer cases were selected. It also lacks an in‐depth analysis of amino acid differences of highly expanded clones and CDR3 between tumor and normal tissues and also lacks timely collection of peripheral blood from the same patient as the experimental group. In addition, we could not find any liver tissue from non‐HBV‐infected liver cancer patients or from healthy donors to use for IR analysis and compare with the TCR repertoire of the tumor and adjacent tumor tissue, and the related literature is also rare to find. Another limitation of the diversity study is that the specific CDR3 sequences were not included. However, the CDR3 overlap rate was used to compare the similarity of TCR clones between and within tumor groups and adjacent normal tissues. We found that there was no significant difference in the similarity between CDR3 in tumor tissues and adjacent cancer groups (Figure S5). The majority of patients with significantly increased or decreased TCR diversity also had high or low CDR3 overlap rates, respectively.^27^, ^38^ The finding that there was no significant difference in similarity (overlap rates) supports that there was no difference in diversity, that is, there was no significant change in diversity after antigen stimulation, which may also indicate that liver tissue is an immune‐privileged organ.^5^, ^39^

In summary, we have comprehensively characterized the TCR repertoires in the tumor and adjacent normal tissues of HCC patients associated with HBV, which will enable a deeper understanding of the T‐cell immune response in HCC patients, further identification of tumor‐specific antigens, and diagnosis and treatment of the disease. In particular, the few differences in the immune repertoire (TRBV CDR3) between tumor and adjacent normal tissue in HCC patients provide a new clue for exploring the mechanism of the liver as an immune‐privileged organ. Although further detailed comparative experiments are needed to confirm the results and the mechanism.

## AUTHOR CONTRIBUTIONS

**Jie‐Zuan Yang:** Conceptualization (lead); investigation (equal); project administration (lead). **Shao‐Yan Xu:** Resources (lead); writing – original draft (equal). **Dang‐Sheng Xiao:** Investigation (equal); methodology (equal). **Jin‐You Li:** Data curation (equal); methodology (equal). **Xiu‐Yuan Jin:** Data curation (equal); methodology (equal). **Dong Yan:** Project administration (equal); resources (equal).

## FUNDING INFORMATION

Zhejiang Province Traditional Chinese Medicine Science and Technology Plan Project (Grant Number: 2023ZL482) and the Natural Science Foundation of Zhejiang Province (Grant Number: LQ23H030005).

## CONFLICT OF INTEREST STATEMENT

No potential conflicts of interest were disclosed.

## Supporting information


Data S1.

## Data Availability

The raw sequence data published in this paper have been deposited in the Genome Sequence Archive of the National Genomics Data Center (NGDC), China National Center for Bioinformation (GSA‐Human: HRA006764), and are publicly available at https://ngdc.cncb.ac.cn/gsa‐human.
