# Supplementary material for: Similar usage of T‐cell receptor β‐chain between tumor and adjacent normal tissue in hepatocellular carcinoma
Source: Cancer Med. 2024 Aug 27;13(16):e70121. doi: 10.1002/cam4.70121 (PMC11349608; doi:10.1002/cam4.70121)
Supplement: Supplementary file 1 — Data S1. [file CAM4-13-e70121-s001.zip › Supplementary files.docx]

Supplementary files

Table

# Table S1 Detail data of TRBV/BJ combination with significant difference between tumor and adjacent normal tissue (Normal) in HCC patients

Please see other document (Excel)

Figures

Fig. 1


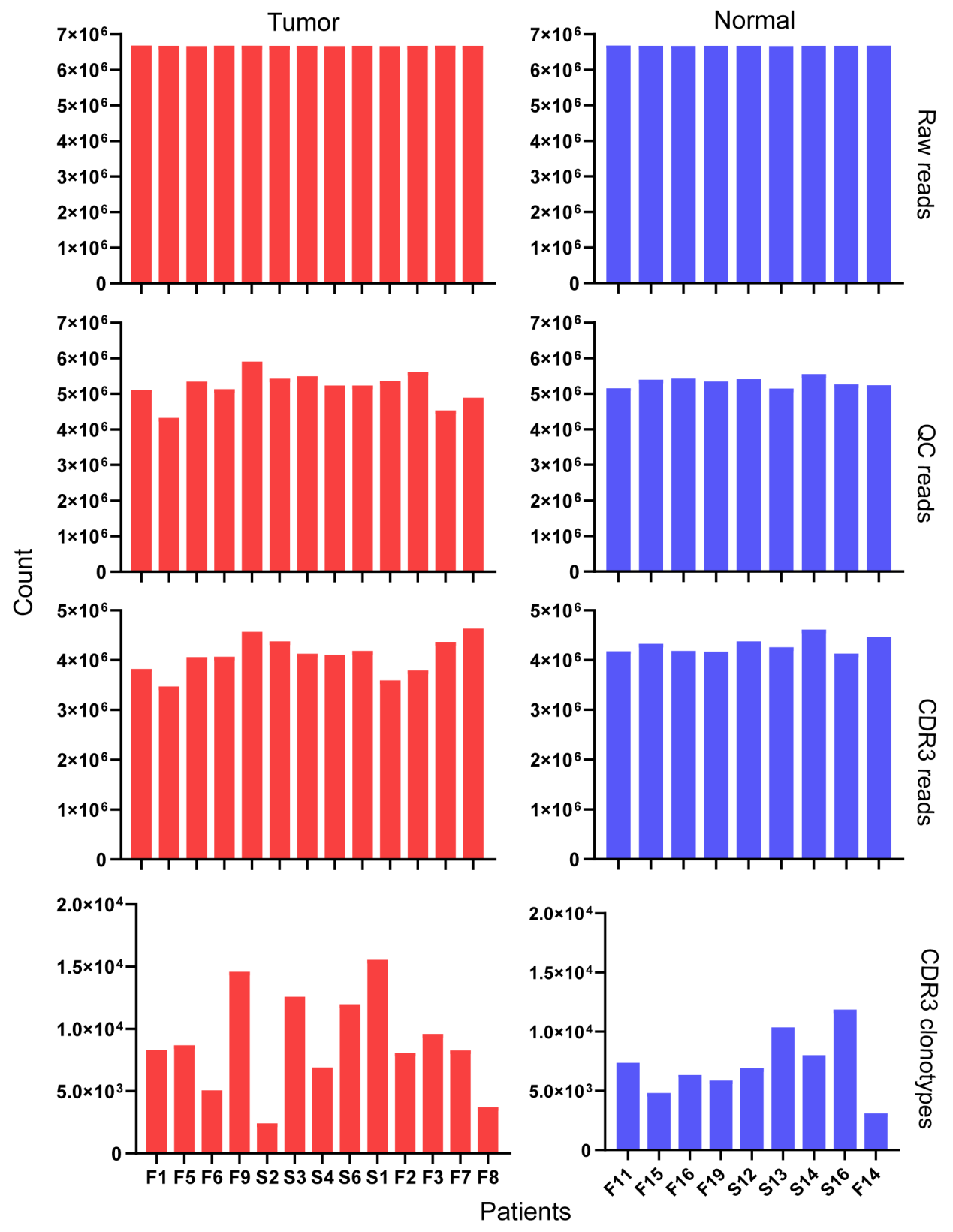


# Figure S**1** Profiling of TCR repertoire sequencing data in each sample

Number of raw reads, QC reads, CDR3 reads, and CDR3 amino acid clonotypes in 13 tumors (left column) and 9 adjacent normal tissues (normal, right column) from 14 patients (8 of whom are paired) with HBV-associated HCC.

TCR, T-cell receptor; QC, quality control; CDR3, complementary determinant region-3

Fig. 2


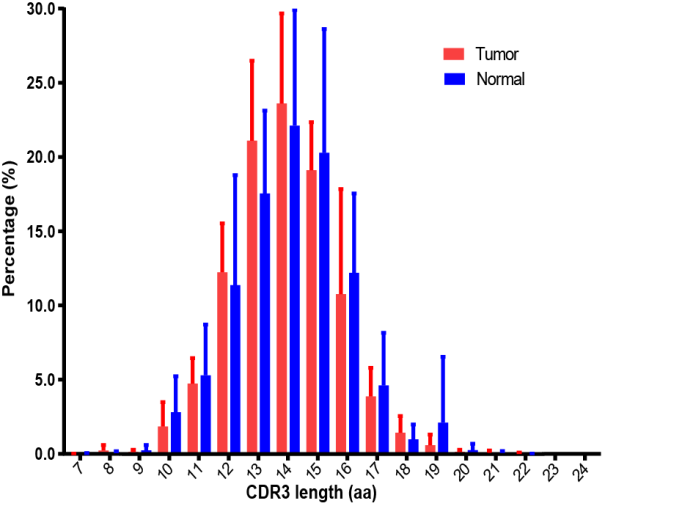


# Figure S**2** Comparison of CDR3aa length distribution in tumor and adjacent normal tissues in HCC patients

Fig. 3


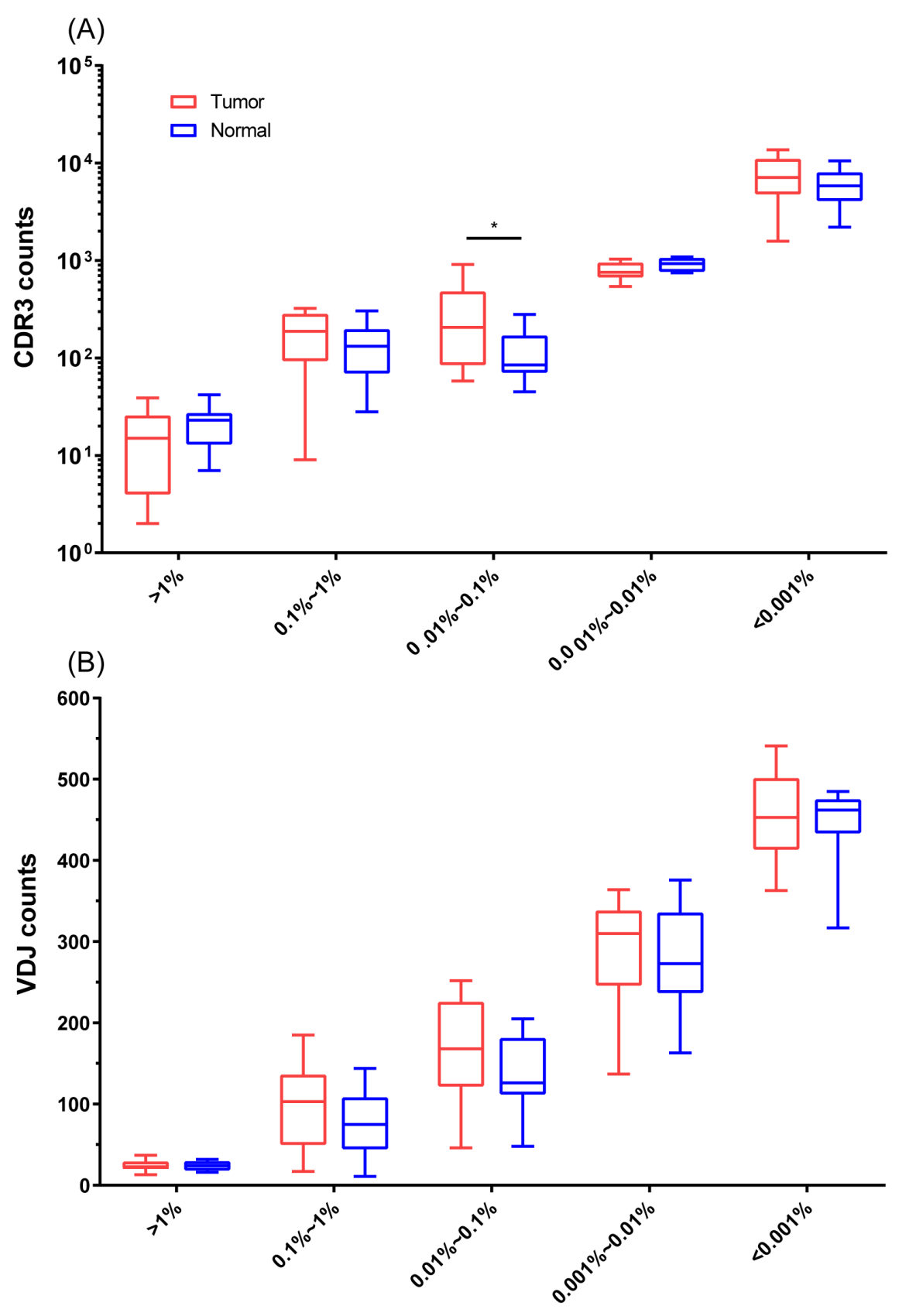


# Figure S**3** Comparison of the number of CDR3 and VDJ numbers based on their different frequency distribution between tumor and adjacent normal tissues

The (A) CDR3 number and (B) VDJ number are each based on different frequency distributions. Data are presented as box and whisker (5–95% percentile) for each patient, with the middle solid line indicating the median CDR3 and VDJ numbers, respectively, and the highest and lowest horizontal lines indicating the 5th and 95th percentiles. P values of the comparisons were calculated using the Mann–Whitney test, *P < 0.05.

Fig. 4


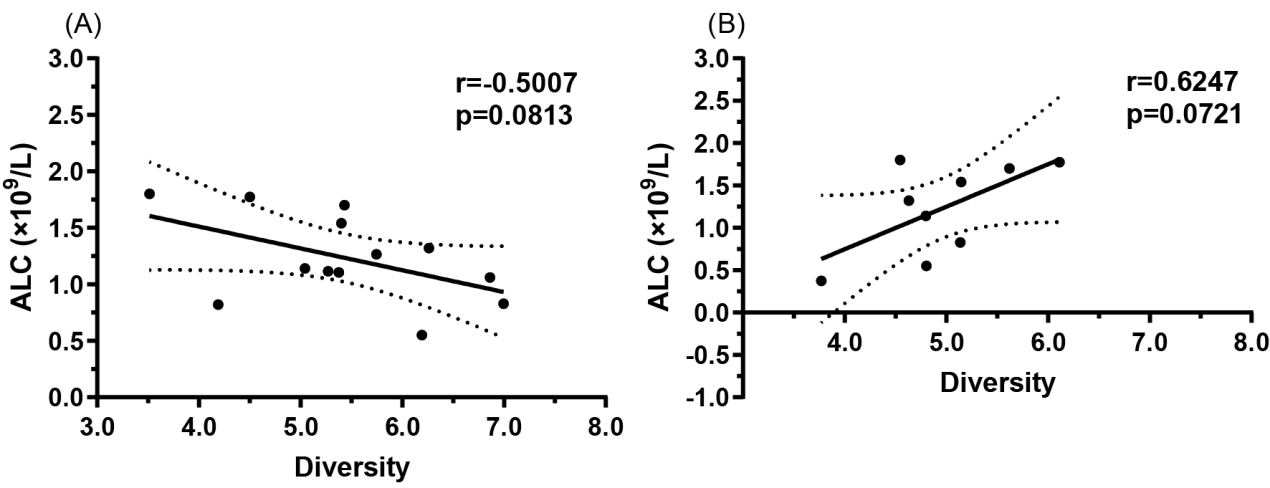


# **Figure S4** **Correlation between absolute lymphocyte count (ALC) and diversity in (A) tumor, in (B) adjacent normal tissue in HCC patients**

Statistical analysis was performed using the Spearman rank test

Fig. 5


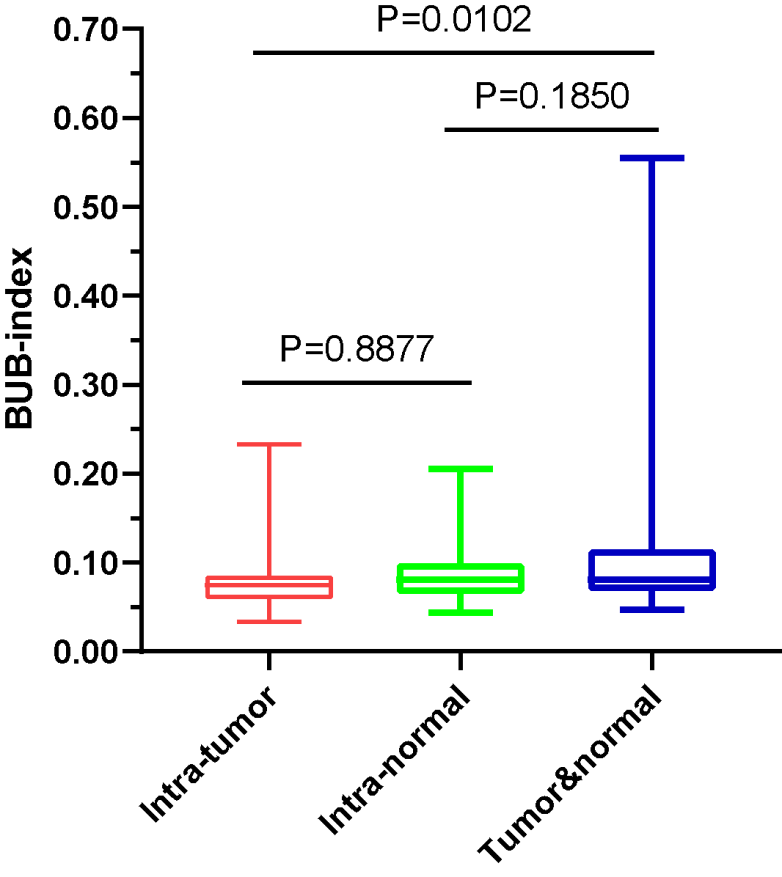


Figure S5 Comparison of BUB index between intra-tumor, intra-normal and tumor&normal

The index of Baroni–Urbani and Buser (BUB) was used as a measure of commonality between TCR sequences. The index BUB ranges from 0 to 1, where 0 represents completely separated populations and 1 represents two populations with complete agreement.
